# Virtual reality modeling for multidisciplinary surgical planning for complex single ventricular physiology

**DOI:** 10.1007/s10554-026-03758-x

**Published:** 2026-06-19

**Authors:** Anokha Nathan, Dhruvil A. Patel, Priya Nair, Michael G. Earing, Stephen Pophal, Madhusudan Ganigara

**Affiliations:** 1 College of Biological Sciences, University of Chicago, 5841 S. Maryland Ave, Chicago, IL 60637 USA; 2 Department of Internal Medicine, University of Chicago Medicine, Chicago, IL USA; 3 Department of Pediatric Cardiology, University of Chicago Medicine, Chicago, IL USA

**Keywords:** Virtual reality, Congenital heart disease, Fontan repair, Single ventricle

## Abstract

The selection of biventricular repair or Fontan completion for single-ventricle patients remains difficult to determine in borderline patients. We report how the use of a 3-dimensional virtual reality model informed an accurate estimation of a 34-month-old child’s ventricular chamber size to confirm Fontan completion. Developed from CT scan imaging, the model simulated a virtual biventricular repair and demonstrated inadequate outflow tracts after patching and baffle rerouting. This case demonstrates the implications of virtual reality for congenital heart disease surgical planning, providing a shared, intuitive platform for multidisciplinary discussion and consensus-building among surgeons, cardiologists, imagers, and anesthesiologists.

## Introduction

The Fontan procedure is the final step of surgical palliation for single-ventricle lesions without a subpulmonary ventricle. After completion, systemic venous return flows passively from the higher-pressure central venous system into the lower-pressure pulmonary circulation. This is an inherently flawed circuit that predisposes recipients to complications from chronically elevated venous pressure, with the most serious being congestive hepatopathy [1].

A biventricular conversion (BiVC) or one-and-a-half ventricle (1.5 V) repair is considered for single-ventricle lesions with “borderline” ventricles of reasonable size, restoring complete or partial anatomical biventricular circulation. Compared with Fontan repair, BiVC recipients developed less, moderate, or greater left ventricular dysfunction (BiV 6.6% vs. Fontan 17.1%, *p* < 0.001) and a lower incidence of liver dysfunction [2]. The decision to pursue BiVC/1.5 V repair relies on an assessment of growth, geometry, and functional reserve of the borderline ventricle, yet the selection criteria remain non-standardized and challenging to apply.

Equally important is the decision not to attempt a conversion when the risk of ventricular inadequacy is high. High-fidelity immersive virtual reality (VR) models permit assessment of chamber size through real-time navigation. VR model metrics are valuable for making these decisions, but they are not well-defined. To highlight relevant paradigms, we present a case of a 34‑month‑old child with a borderline right ventricle (RV) evaluated for possible BiVC/1.5 V repair. Decision-making was aided by VR modeling based on high‑resolution cardiac CT, which became pivotal in deciding to proceed with Fontan palliation rather than BiVC/1.5 V repair.

## Case presentation

A 34‑month‑old male with hypoplastic RV, D‑transposition of the great arteries, ventricular septal defect, and pulmonary stenosis underwent a right‑sided systemic-to-pulmonary shunt followed by a right‑sided bidirectional Glenn procedure. The RV was bipartite with a hypoplastic trabecular component.

**Fig. 1:**
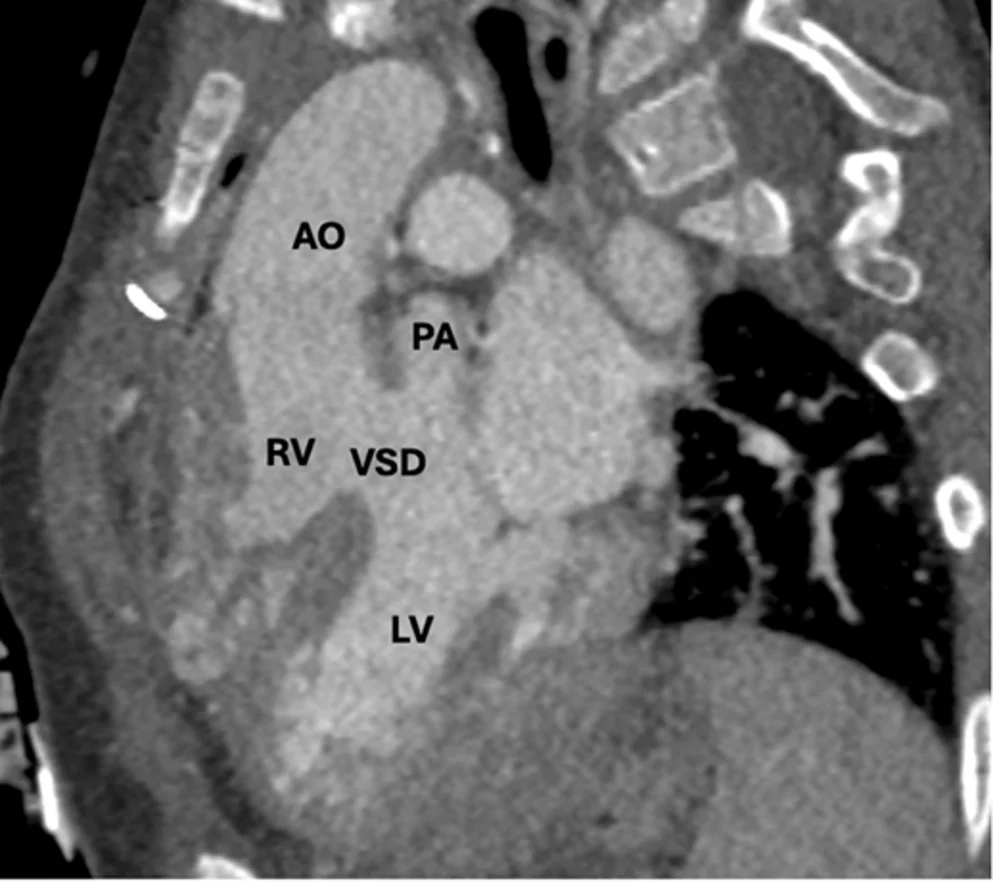
Cardiac MRI sagittal view of ventricular septal defect with a distinct small right ventricular chamber. (AO = Aorta, PA = Pulmonary Artery, RV = Right Ventricle, VSD = Ventricular Septal Defect, LV = Left Ventricle)

For Fontan consideration, cardiac MRI showed interval RV growth with normal RV volumes (RV end‑diastolic volume 80 mL/m²), qualitative hypertrophy, and normal systolic function (RV ejection fraction 52%) (Figure 1). The left ventricle (LV) was moderately dilated (LV end‑diastolic volume 132 mL/m²) with low‑normal systolic function (LV ejection fraction 50%). A contrast-enhanced cardiac CT was obtained to further define the anatomy. Using Elucis™ (*Realize Medical™*,* Ottawa*,* ON*), the 3D CT dataset was manually modeled using Meta Quest Pro*™* (*Meta Platforms*^®^,* Inc.*) goggles. Pre‑operative cardiac catheterization demonstrated hemodynamics suitable for Fontan completion.

**Fig. 2:**
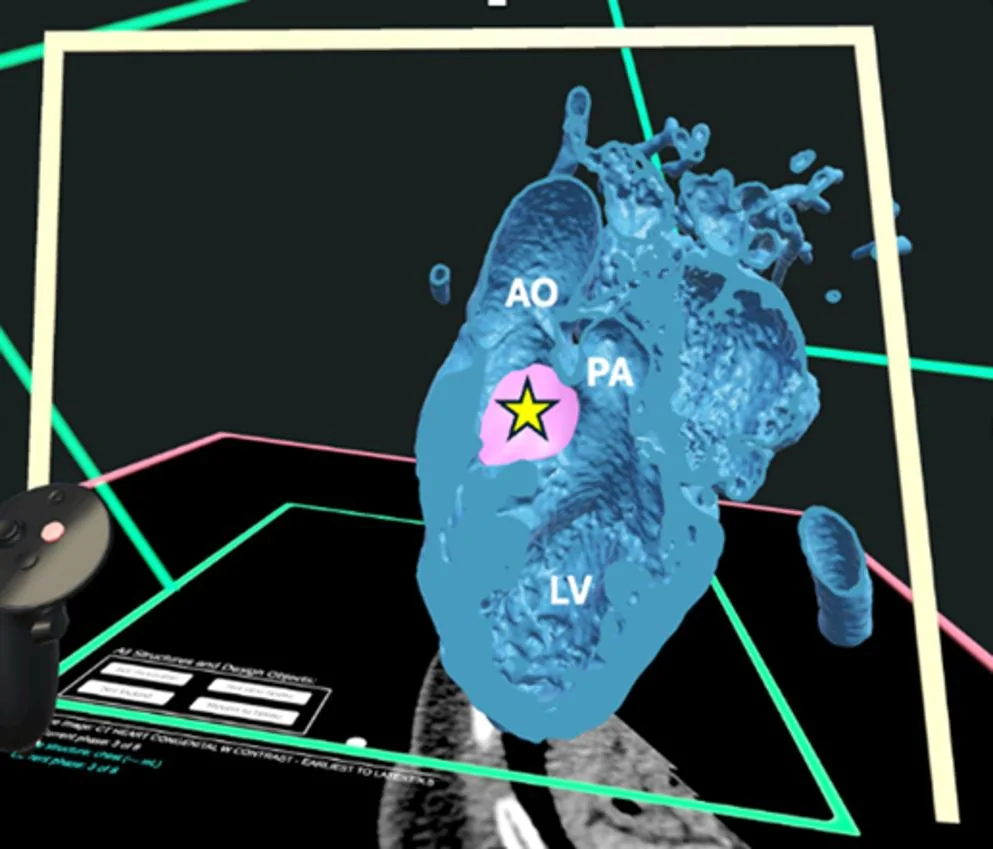
Virtual reality model reconstructed for CT shown in Fig. 1 within Elucius™ from Realize Medical and visualized using Meta Quest Pro 3 device. The star on the model indicates the site for VSD patch implementation (pink area), showing the LV baffle to the aorta for surgical planning. (AO = Aorta, PA = Pulmonary Artery, LV = Left Ventricle)

At the multidisciplinary surgical conference, VR cardiac anatomy was jointly reviewed. A virtual VSD patch/LV–aorta baffle was constructed in the patient’s VR model (Figure 2). This “virtual repair” demonstrated that the patch would extend across the RV toward the D‑malposed aorta, significantly encroaching on the functional RV cavity and further reducing an already borderline RV volume. The consensus was that, although MRI-derived RV volumes were reliable, the effective postoperative RV size and geometry would likely be inadequate once the baffle was in place. The team therefore elected to proceed with an extracardiac Fontan, which the patient tolerated with good postoperative recovery.

## Discussion

While imaging suggested RV volumes were within acceptable limits, visualization in the immersive VR environment—combined with virtual patch and baffle creation—made the non-feasibility of repair given the RV geometry and volumes visually evident. Beyond static 3D printing, VR allows surgeons and cardiologists to interact with patient‑specific anatomy at true scale, simulate surgical maneuvers (such as baffle routing or VSD patch placement), and immediately appreciate trade‑offs in chamber size, inflow/outflow alignment, and risk of obstruction. Virtual baffle creation, patch sizing, and conduit positioning are rapidly becoming part of the planning workflow for complex biventricular conversions, double‑outlet ventricle repairs, and re‑operations in patients with prior complex surgeries.

Long-term outcomes are comparable between biventricular and single-ventricular approaches, but a biventricular approach mitigates the almost definite long-term complications of single-ventricular physiology [2]. 3D and VR technologies provide an innovative solution to the dilemma of decision-making in these complex cases. VR enables visualization of chamber volumes at a 1:1 scale to underpin decisions, bypassing assumptions made in processing imaging to create mental models.

There is a dearth of prospective data on when Fontan repair is appropriate compared with BiCV repair for borderline patients, for which suggested frameworks are based on retrospective series and institutional experience [3]. Immersive VR may provide an intuitive visual assessment without relying on metrics and measurements, with advanced models simulating real-time function. When VR models of patient anatomy BiVC recipients prior to repair was presented to pediatric cardiac surgeons, surgeon concordance with the surgical approach performed was greatest with the VR model compared with static images or a 3D-printed model [4]. Representing VR models as an accurate adjuvant for surgical intuition – especially in cases where clear metrics cannot inform approach. Yet reporting and model quality is highly variable across operators, underscoring the need for standardized technical and clinical evaluation frameworks to support workflows [5].

## Conclusion

3D and VR technologies are increasingly being integrated into preoperative planning for complex congenital heart surgery. Our case adds to growing evidence that VR modeling and virtual repair planning can meaningfully influence surgical strategy in complex congenital heart disease. A decision not to pursue biventricular or 1.5 V repair has major lifelong implications. As these tools become more widely available and standardized, VR‑based planning is likely to become the norm rather than the exception in high‑complexity CHD surgery.

## Data Availability

No datasets were generated or analysed during the current study.
